# Nonoscillatory Central Schemes for Hyperbolic Systems of Conservation Laws in Three-Space Dimensions

**DOI:** 10.1155/2013/672187

**Published:** 2013-08-18

**Authors:** Andrew N. Guarendi, Abhilash J. Chandy

**Affiliations:** Department of Mechanical Engineering, The University of Akron, Akron, OH 44325-3903, USA

## Abstract

We extend a family of high-resolution, semidiscrete central schemes for hyperbolic systems of conservation laws to three-space dimensions. Details of the schemes, their implementation, and properties are presented together with results from several prototypical applications of hyperbolic conservation laws including a nonlinear scalar equation, the Euler equations of gas dynamics, and the ideal magnetohydrodynamic equations. Parallel scaling analysis and grid-independent results including contours and isosurfaces of density and velocity and magnetic field vectors are shown in this study, confirming the ability of these types of solvers to approximate the solutions of hyperbolic equations efficiently and accurately.

## 1. Introduction

Over the past couple of decades, much work has gone into the construction, analysis, and implementation of modern numerical algorithms for the approximate solution of systems of nonlinear hyperbolic conservation laws of the form

(1) $$\begin{array}{c} {\left(u\right)}_{t}+f{\left(u\right)}_{x}+g{\left(u\right)}_{y}+h{\left(u\right)}_{z}=0. \end{array}$$

Numerical solutions of these equations are of tremendous practical importance as they govern a variety of physical phenomena in natural and engineering applications. In particular, a number of high-resolution schemes have been developed and tested for this purpose [1–3]. The first-order Lax-Friedrichs scheme [4] is actually the forerunner for a large class of central high-resolution schemes that have seen much development in recent years. This paper presents the formulation and testing of such 3D central high-resolution schemes.

These central schemes enjoy a major advantage of simplicity over methods like upwind schemes, in that no approximate Riemann solvers are involved in their construction. To provide a brief background, in 1990, Nessayahu and Tadmor introduced a fully discrete second-order nonoscillatory central scheme (NT scheme), [1], which was further extended to higher orders of accuracy [5–7], as well as to multidimensional systems [8–10]. The main ingredient in the construction of the NT method was a second-order nonoscillatory, monotonic upstream scheme for conservation laws-(MUSCL-) type [11], piecewise linear interpolant (instead of the piecewise constant one employed in the Lax-Friedrichs scheme) in combination with a higher-order solver for the time evolution [1]. However, applying the fully discrete NT scheme to the second-order convection-diffusion equations did not provide the desired resolution of discontinuities (see [12–14]) due to the accumulation of excessive numerical dissipation [12, 15]. This led Kurganov and Tadmor [12] to the development of a set of second-order semi-discrete central schemes, which had smaller dissipation than the original NT scheme, and, unlike the fully discrete central schemes, they could be efficiently used with time steps as small as required by the CFL stability restriction due to the diffusion term.

The schemes presented in Kurganov and Tadmor's study saw further developments in the form of third-order extensions [15] and central weighted essentially nonoscillatory (CWENO) reconstruction [8] (also see [16, 17] for essentially nonoscillatory (ENO) implementations). Such weighted essentially nonoscillatory (WENO) reconstructions were introduced first in an upwind framework [18] after which they were extended to a central framework [5, 8, 9, 19]. More recently Balbás and Tadmor [20, 21] presented extensions of the semidiscrete central schemes of [12] with arbitrary order, *r*, specifically third- and fourth-order reconstructions with the possibility of additional reconstructions in the diagonal directions. In this paper, we present and test the third-order accurate semidiscrete central schemes of Balbás and Tadmor [21] for 3D systems of equations.

## 2. Formulation

**Figure 1:**
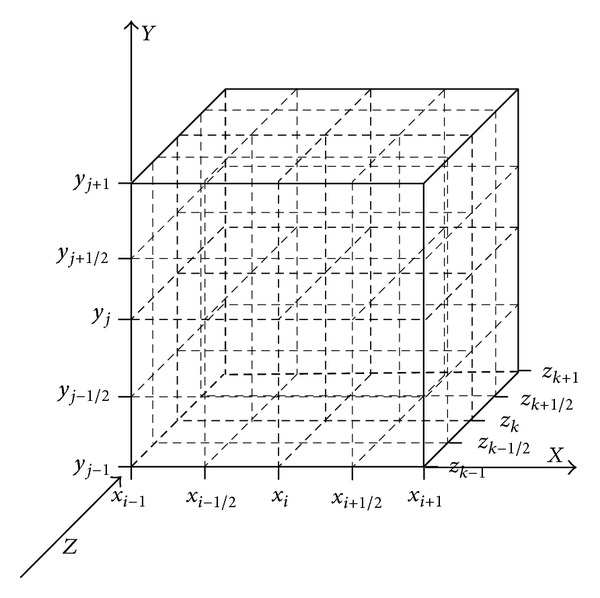
Modified central differencing in three-space dimensions.

Starting with a general hyperbolic conservation law in three-space dimensions, (1), let the sliding averages of *u* over the cells *C*_*i*,*j*,*k*_ : = [*x*_*i*−1/2_, *x*_*i*+1/2_]×[*y*_*j*−1/2_, *y*_*j*+1/2_]×[*z*_*k*−1/2_, *z*_*k*+1/2_] (see Figure 1) at time level, *n*, be

(2) $$\begin{array}{c} {\overset{-}{u}}_{i,j,k}^{n}:=\frac{1}{\Delta x\Delta y\Delta z}\overset{}{\underset{C_{i,j,k}}{\int }}u\left(x,y,z,t^{n}\right)\;dx\;dy\;dz, \end{array}$$

where Δ*x*, Δ*y*, and Δ*z* are the cell widths in the *x*-, *y*-, and *z*-directions, respectively.

**Figure 2:**
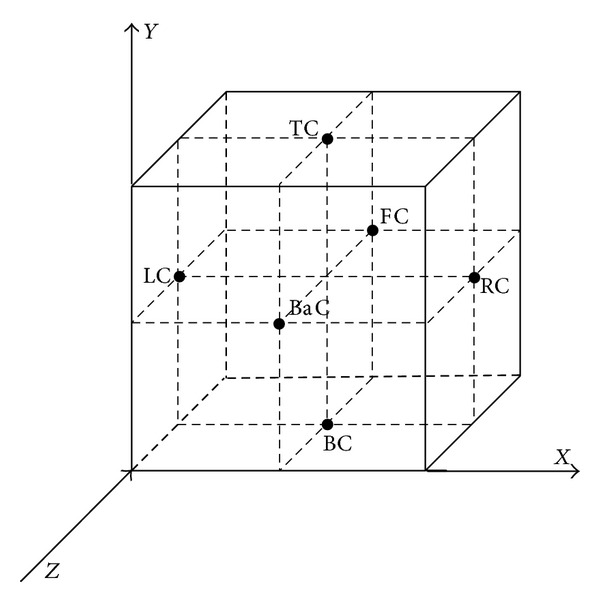
Reconstructions in the *x*-, *y*-, and *z*-directions.

Following [21], the first step passing from a fullydiscrete to a semi-discrete formulation consists of reducing the size of the staggered cells covering the Riemann fans. So cells with variable width of order *O*(Δ*t*) are used, by incorporating the maximum local speeds of propagation across the cell interfaces [12, 22], which are given by

(3) $$\begin{array}{c} a_{i+1/2,j,k}^{n}:=\;\max\left[\rho \left(\frac{\partial f}{\partial u}\left(u_{i+1,j,k}^{\text{LC}}\right)\right),\rho \left(\frac{\partial f}{\partial u}\left(u_{i,j,k}^{\text{RC}}\right)\right)\right],\\ b_{i,j+1/2,k}^{n}:=\;\max\left[\rho \left(\frac{\partial g}{\partial u}\left(u_{i,j+1,k}^{\text{BC}}\right)\right),\rho \left(\frac{\partial g}{\partial u}\left(u_{i,j,k}^{\text{TC}}\right)\right)\right],\\ c_{i,j,k+1/2}^{n}:=\;\max\left[\rho \left(\frac{\partial h}{\partial u}\left(u_{i,j,k+1}^{\text{BaC}}\right)\right),\rho \left(\frac{\partial h}{\partial u}\left(u_{i,j,k}^{\text{FC}}\right)\right)\right], \end{array}$$

where the superscripts LC, RC, BC, TC, BaC and FC stand for the left, right, bottom, top, back, and front centers, respectively (Figure 2). The previous equations are exact for genuinely nonlinear and linearly degenerate fields [21].

The cell interface values in the *x*-, *y*-, and *z*-directions, shown in the previous equations, are defined as

(4) $$\begin{array}{c} u_{i,j,k}^{\text{RC}}:=p_{i,j,k}^{n}\left(x_{i+1/2},y_{j},z_{k}\right),\\ u_{i,j,k}^{\text{LC}}:=p_{i,j,k}^{n}\left(x_{i-1/2},y_{j},z_{k}\right),\\ u_{i,j,k}^{\text{TC}}:=p_{i,j,k}^{n}\left(x_{i},y_{j+1/2},z_{k}\right),\\ u_{i,j,k}^{\text{BC}}:=p_{i,j,k}^{n}\left(x_{i},y_{j-1/2},z_{k}\right),\\ u_{i,j,k}^{\text{FC}}:=p_{i,j,k}^{n}\left(x_{i},y_{j},z_{k+1/2}\right),\\ u_{i,j,k}^{\text{BaC}}:=p_{i,j,k}^{n}\left(x_{i},y_{j},z_{k-1/2}\right). \end{array}$$

They are calculated via a nonoscillatory piecewise polynomial reconstruction given by

(5) $$\begin{array}{c} R\left(x,y,z;{\overset{-}{u}}^{n}\right)=\sum_{i,j,k}p_{i,j,k}^{n}\left(x,y,z\right)1{}_{C_{i,j,k}}; \end{array}$$

the polynomials *p*_*i*,*j*,*k*_^*n*^(*x*, *y*, *z*) are determined so that $R(x,y,z;{\overset{-}{u}}^{n})$ satisfies properties such as conservation of cell averages, accuracy, and nonoscillatory behavior (see [21]). For more details about the derivation of the polynomials and their properties, see [21]. This information allows the separation of regions of smoothness and regions of nonsmoothness, where the discontinuities propagate in one or more directions. In the nonsmooth regions, staggered evolution over the respective control volumes is used to obtain cell averages, while, for the smooth regions, the polynomial *p*_*i*,*j*,*k*_^*n*^(*x*, *y*, *z*) and the corresponding fluxes are integrated over the respective control volume to obtain cell averages in these regions. This way, a new polynomial, say $R\left(x,y,z;{\overset{-}{w}}^{n}\right)$, is formed, which is reconstructed from these smooth and non-smooth portions of the solution and it is reprojected back onto the original grid cells.

The resulting semi-discrete scheme in the limit as Δ*t* → 0 is as follows:

(6) ddtu−i,j,k(t)=−Hi+1/2,j,kx(t)−Hi−1/2,j,kx(t)Δx −Hi,j+1/2,ky(t)−Hi,j−1/2,ky(t)Δy −Hi,j,k+1/2z(t)−Hi,j,k−1/2z(t)Δz

with numerical fluxes

(7) Hi+1/2,j,kx(t)=12[f(ui+1,j,kLC)+f(ui,j,kRC)]      −ai+1/2,j,k(t)2[ui+1,j,kLC−ui,j,kRC],Hi,j+1/2,ky(t)=12[g(ui,j+1,kTC)+g(ui,j,kBC)]      −bi,j+1/2,k(t)2[ui,j+1,kTC−ui,j,kBC],Hi,j,k+1/2z(t)=12[h(ui,j,k+1FC)+h(ui,j,kBaC)]      −ci,j,k+1/2(t)2[ui,j,k+1FC−ui,j,kBaC]

for the third-order CWENO reconstruction without any diagonal smoothing (diagonal smoothing described in Section 3). There is also an option of carrying out diagonal smoothing, the equations of which are not provided here.

## 3. Implementation of Semidiscrete Central Schemes: Cell Interface Reconstructions

This section provides a third-order nonoscillatory reconstruction in three-space dimensions, that was implemented for computing the solutions of hyperbolic conservation laws (see (19), (21), and (22)–(25)). The reconstruction of the point values of *u* presented in this section is the third-order CWENO polynomial reconstruction of Kurganov and Levy [15]. The properties of the piecewise quadratic polynomial, that is used here, were presented in [15, 21]. In each cell *C*_*i*,*j*,*k*_ (defined before), the polynomials [*p*_*i*,*j*,*k*_^*n*^(*x*, *y*, *z*)] in (4) are written as a convex combination of three polynomials *P*_−1_(*x*, *y*, *z*), *P*_0_(*x*, *y*, *z*), and *P*_1_(*x*, *y*, *z*). In the *x*-direction they are as follows:

(8) Pi,j,kn(x,y,z)=w−1P−1(x,y,z)+w0P0(x,y,z) +w1P1(x,y,z), ∑m∈−1,0,1wm=1,

where the linear polynomials

(9) $$\begin{array}{c} P_{-1}\left(x,y,z\right)={\overset{-}{u}}_{i,j,k}^{n}+\frac{{\overset{-}{u}}_{i,j,k}^{n}-{\overset{-}{u}}_{i-1,j,k}^{n}}{\Delta x}\left(x-x_{i}\right),\\ P_{1}\left(x,y,z\right)={\overset{-}{u}}_{i,j,k}^{n}+\frac{{\overset{-}{u}}_{i+1,j,k}^{n}-{\overset{-}{u}}_{i,j,k}^{n}}{\Delta x}\left(x-x_{i}\right) \end{array}$$

conserve the pair of cell averages ${\overset{-}{u}}_{i-1,j,k}^{n}$, ${\overset{-}{u}}_{i,j,k}^{n}$ and ${\overset{-}{u}}_{i,j,k}^{n}$, ${\overset{-}{u}}_{i+1,j,k}^{n}$, respectively, and the parabola centered around *x*_*i*_,

(10) P0(x,y,z)=u−i,j,kn−112((u−i−1,j,kn−2u−i,j,kn+u−i+1,j,kn)      +(u−i,j,k−1n−2u−i,j,kn+u−i,j,k+1n)) +u−i+1,j,kn−u−i−1,j,kn2Δx(x−xi) +u−i+1,j,kn−u−i,j,kn+u−i−1,j,knΔx2(x−xi)2,

is determined so as to satisfy

(11) c−1P−1(x,y,z)+c1P1(x,y,z)   +(1−c−1−c1)P0(x,y,z)  =ui,j,kn+ux ∣ i,j,k(x−xi)+12uxx ∣ i,j,k(x−xi)2,

where

(12) ui,j,kn≔u−i,j,kn−124((u−i−1,j,kn−2u−i,j,kn+u−i+1,j,kn)       +(u−i,j−1,kn−2u−i,j,kn+u−i,j+1,kn)       +(u−i,j,k−1n−2u−i,j,kn+u−i,j,k+1n)),ux ∣ i,j,k≔u−i+1,j,kn−u−i−1,j,kn2Δx,uxx ∣ i,j,k≔u−i+1,j,kn−u−i,j,kn+u−i−1,j,knΔx2.

Note that for the central parabola equation (10) additional corrections in the *y*- and *z*-directions guarantee third-order accuracy when the point value *u*(*x*_*i*_, *y*_*j*_, *z*_*k*_, *t* ^*n*^) is recovered from the neighboring cell averages.

The conservation of the cell averages ${\overset{-}{u}}_{i-1,j,k}^{n},{\overset{-}{u}}_{i,j,k}^{n},{\overset{-}{u}}_{i+1,j,k}^{n}$ and the accuracy property (property **P**_2_ in [8, 13]) are guaranteed [8, 21] by any symmetric choice of weights *c*_*m*_ (e.g., *c*_−1_ = *c*_1_ = 1/4, *c*_0_ = 1/2). The nonoscillatory behavior (property **P**_3_ in [8, 21]) is attained with nonlinear weights

(13) $$\begin{array}{c} w_{m}=\frac{\alpha_{m}}{\sum_{l}\alpha_{l}},\;\text{with}\;\alpha_{m}=\frac{c_{m}}{{\left(\epsilon +IS_{m}\right)}^{2}},\;m,l\in \left\{-1,0,1\right\}\text{.} \end{array}$$

*ϵ* ≪ 1 prevents the denominator from being zero (*ϵ* = 10^−6^), and the smoothness indicators provide a local measure of the derivatives *P*_*m*_(*x*, *y*, *z*), switching automatically to the second-order reconstructions *P*_−1_ and *P*_1_ in the presence of steep gradients and avoiding the onset of spurious oscillations [9, 15, 18], and in this case they read

(14) IS−1=(u−i,j,kn−u−i−1,j,kn)2,IS1=(u−i+1,j,kn−u−i,j,kn)2,IS0=133(u−i+1,j,kn−2u−i,j,kn+u−i−1,j,kn)2, +14(u−i+1,j,kn−u−i−1,j,kn)2.

In the case of systems of equations, the smoothness indicators are given by the *norm-scaled* average of the componentwise indicators, *IS*_*m*_^(*nq*)^, given by

(15) $$\begin{array}{c} IS_{m}=\frac{1}{EQ}\overset{EQ}{\underset{nq=1}{\sum }}\frac{1}{{||u^{\left(nq\right)}||}_{2}+\epsilon }IS_{m}^{\left(nq\right)},\;m\in \left\{-1,0,1\right\}, \end{array}$$

where *u* ^(*nq*)^ stands for the (*nq*)th component of *u* and

(16) $$\begin{array}{c} {||u^{\left(nq\right)}||}_{2}=\underset{i,j,k}{\sum }{\left|u_{i,j,k}^{\left(nq\right)}\right|}^{2}\Delta x\Delta y\Delta z \end{array}$$

represents its *l*_2_ norm over the discretized solution domain.

The interface values can now be calculated from (8)–(10) as follows:

(17) ui,j,kRC:=pi+1/2,j,kn(x,y,z)=w−1[u−i,j,kn+12(u−i,j,kn−u−i−1,j,kn)] +w0[u−i,j,kn−112((u−i−1,j,kn−2u−i,j,kn+u−i+1,j,kn)        +(u−i,j−1,kn−2u−i,j,kn+u−i,j+1,kn)        +(u−i,j,k−1n−2u−i,j,kn+u−i,j,k+1n))    +14(u−i+1,j,kn−u−i−1,j,kn)    +14(u−i+1,j,kn−2u−i,j,kn+u−i−1,j,kn)] +w1[u−i,j,kn+12(u−i+1,j,kn−u−i,j,kn)],ui,j,kLC:=pi−1/2,j,kn(x,y,z)=w−1[u−i,j,kn−12(u−i,j,kn−u−i−1,j,kn)] +w0[u−i,j,kn−112((u−i−1,j,kn−2u−i,j,kn+u−i+1,j,kn)        +(u−i,j−1,kn−2u−i,j,kn+u−i,j+1,kn)        +(u−i,j,k−1n−2u−i,j,kn+u−i,j,k+1n))    −14(u−i+1,j,kn−u−i−1,j,kn)    +14(u−i+1,j,kn−2u−i,j,kn+u−i−1,j,kn)] +w1[u−i,j,kn−12(u−i+1,j,kn−u−i,j,kn)].

Similar reconstructions to (8) can be carried out in the *y*- and *z*-directions and the interface values *u*_*i*,*j*,*k*_^FC^, *u*_*i*,*j*,*k*_^BaC^, *u*_*i*,*j*,*k*_^TC^, and *u*_*i*,*j*,*k*_^BC^ can be derived in a straightforward manner.  Section 4 presents specific test cases related to the governing equations presented before along with a parallel scaling analysis of the implementation of such schemes on multiple platforms.

## 4. Numerical Test Cases

This section presents results from the solutions of a single scalar equation (19), the Euler equations of gas dynamics (21), and the ideal equations of magnetohydrodynamics (22) and systems of equations and in applications of a scalar convection problem, the Richtmyer-Meshkov instability, and the Orszag-Tang vortex problem, respectively. For all the calculations presented here, we choose a uniform grid in physical space. For temporal discretization, the third-order strong stability-preserving (SSP) Runge-Kutta [23, 24] is used and the time step is dynamically calculated to satisfy the CFL restriction given by

(18) Δt=CFL×((max⁡|ai,j,k|)2(Δx)2+(max⁡|bi,j,k|)2(Δy)2+(max⁡|ci,j,k|)2(Δz)2)−1,

where *a*_*i*,*j*,*k*_, *b*_*i*,*j*,*k*_, and *c*_*i*,*j*,*k*_ are the speeds of propagation (3). The CFL number was chosen to be 0.5 in all the cases presented here.

### 4.1. Single Scalar Equation: Inviscid Burgers Equation

The presentation of three-dimensional semi-discrete schemes, formulated in Section 3, begins with the solution of a single scalar equation given by

(19) $$\begin{array}{c} {\left(u\right)}_{t}+\nabla \cdot \left(u^{2}\right)=0. \end{array}$$

The equation is solved in a 3D computational domain of size 1 × 1 × 1 with a total of up to 100^3^ points, and the initial conditions are such that the variable *u* is given by

(20) $$\begin{array}{c} u=0.5;\;0.1\leq x\leq 0.5,\;0.1\leq y\leq 0.5,\;0.1\leq z\leq 0.5, \end{array}$$

and zero elsewhere.

**Figure 3:**
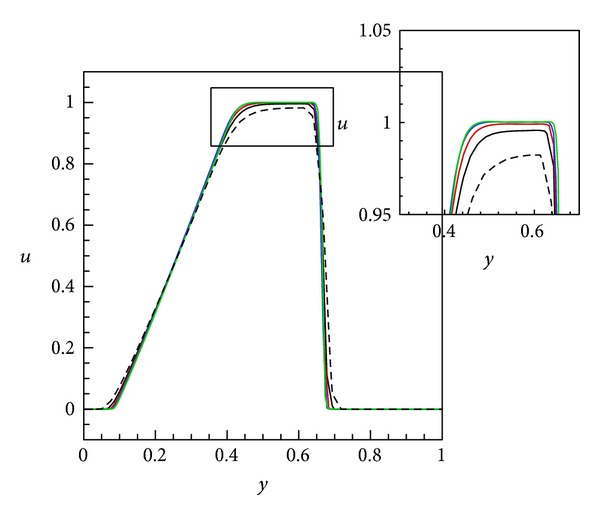
Instantaneous solutions, at *X* = 0.5 and *Y* = 0.5, of the nonlinear scalar equation (19) using the CWENO polynomial reconstruction at time *t* = 0.2 for various grid resolutions. Lines: dashed black :40^3^, solid black :60^3^, solid red :80^3^, solid blue :100^3^, and solid green :120^3^.

Firstly, the solution of the previous equation is presented at various grids to assess the convergence of such a scheme. Also note that no diagonal smoothing is applied in this case. In Figure 3, solutions are obtained at various grid resolutions such as 40^3^, 60^3^, 80^3^, 100^3^, and 120^3^. The solution with 40^3^ resolution is highly inaccurate due to excessive dissipation and can be disregarded in this case. As we go from 60^3^ to 120^3^, the solution starts to improve, and eventually, it does not change; that is, the difference between the 100^3^and 120^3^ solutions is not significant. This also indicates that the solution becomes grid independent.

**Figure 4:**
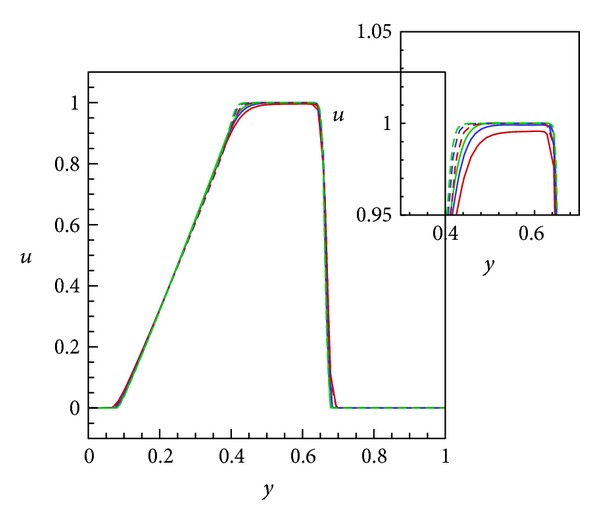
Instantaneous solutions, at *X* = 0.5 and *Y* = 0.5, of the nonlinear scalar equation (19) at time *t* = 0.2 for 3rd- and 2nd-order CWENO reconstructions and at various grid resolutions. Solid lines: 3rd order, dashed lines: 2nd order, red: 60^3^, blue: 80^3^, and green: 100^3^.

Next, the schemes presented in this paper are compared to the second-order scheme of Kurganov and Tadmor [12], where a nonoscillatory reconstruction consisting of a minmod limiter was used.  Figure 4 shows the 3rd- and 2nd-order solutions at three different grid resolutions in order to assess if there is in fact an improvement in resolution with the order of the scheme. With regard to resolving the discontinuities, the 3rd-order scheme presented here exhibits slightly more dissipation than the 2nd-order method at all resolutions. The solution is expected to dissipate more with the application of diagonal smoothing.

**Figure 5:**
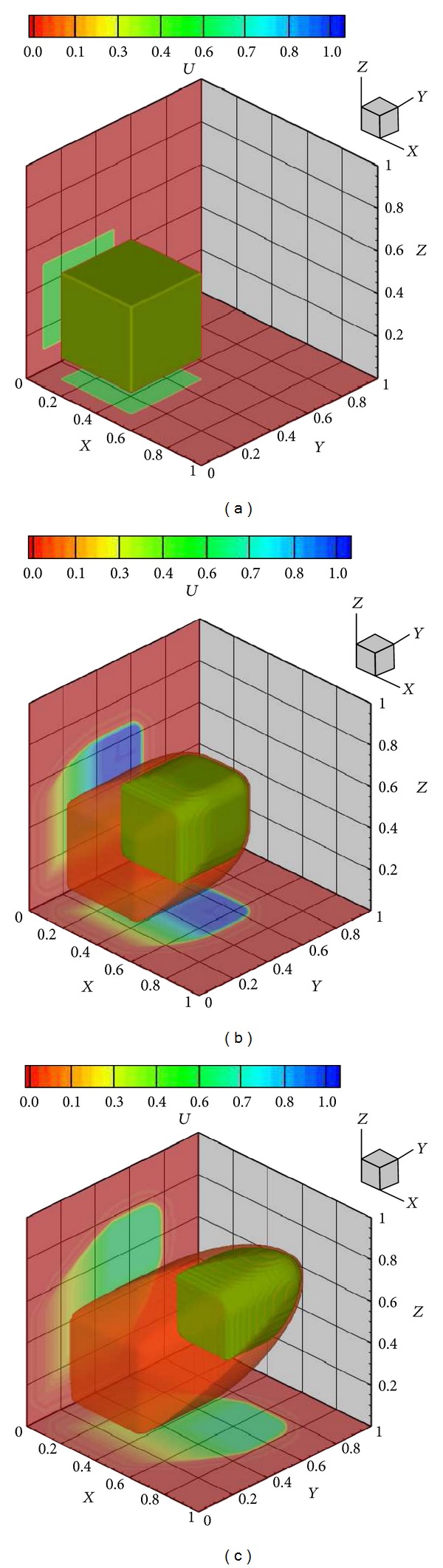
Instantaneous solutions of the nonlinear scalar equation (19) using the CWENO polynomial reconstruction diagonal smoothing at times (a) *t* = 0, (b) *t* = 0.4, and (c) *t* = 0.8. Shown are isosurfaces at two values 0.5 and 0.05, along with slices along *X* = 0.3 and *Z* = 0.3, which are projected to the end of the domain. Initial condition—*u* = 0.5, for 0.1 ≤ *x* ≤ 0.5, 0.1 ≤ *y* ≤ 0.5, and 0.1 ≤ *z* ≤ 0.5.

Figure 5 presents the entire 3D solution of (19) using the polynomial reconstructions *without* diagonal smoothing, respectively, for 100^3^ grid resolution. Isosurfaces at two different values and the slices in two different directions are shown at various times. The nonzero region or “cube” moves towards the corner (1,1, 1) and diffuses as well, as time progresses.

### 4.2. Parallel Performance Analysis for System of Equations

**Figure 6:**
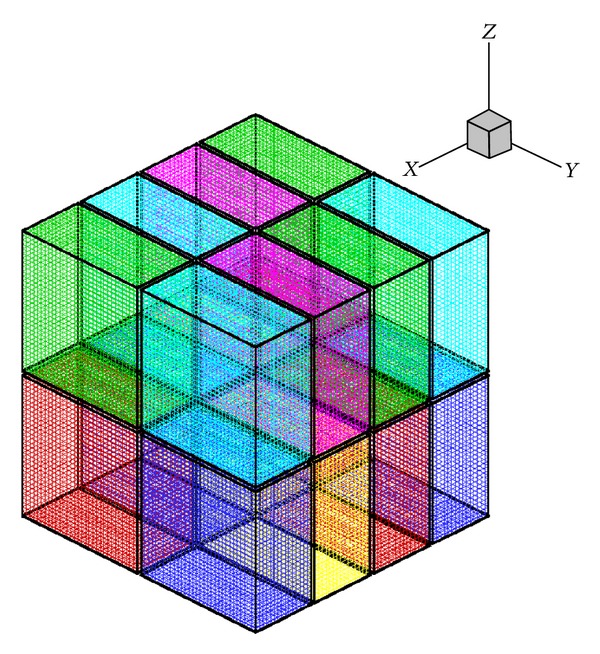
Mesh and domain decomposition for 64 × 64 × 64 grid with a 4 × 2 × 2 processor configuration.

The computational requirements for the solution of hyperbolic problems could become prohibitive in the case of three-dimensional, geometrically complex enclosures. These requirements increase further when realistic fluid flows like viscous or turbulent flows are considered, thereby requiring larger computational effort and memory. Recent developments in high-performance computing promise a substantial increase in computational speed and offer new possibilities for more accurate simulations. Three-dimensional domain decomposition is used to speed the calculations, where the computational domain is decomposed into a number of rectangular blocks with each processor being responsible for a single block. An example of this decomposition can be seen by the gaps in the grid in Figure 6 for the specific case of 16 processors.

Most of the calculations in the interior of each of the subdomains are independent of the domain decomposition and can continue as if they are performed serially. Problems arise near the subdomain boundaries where, for example, finite differences calculated adjacent to the subdomain boundaries may need several points outside the subdomain. To support these circumstances, two rows of “ghost points” are carried along with the interior solutions that contain copies of the interior solution from the neighboring subdomain. These points are exchanged and updated from neighboring processors as needed to ensure that all near-wall calculations are performed with current variable values.

**Figure 7:**
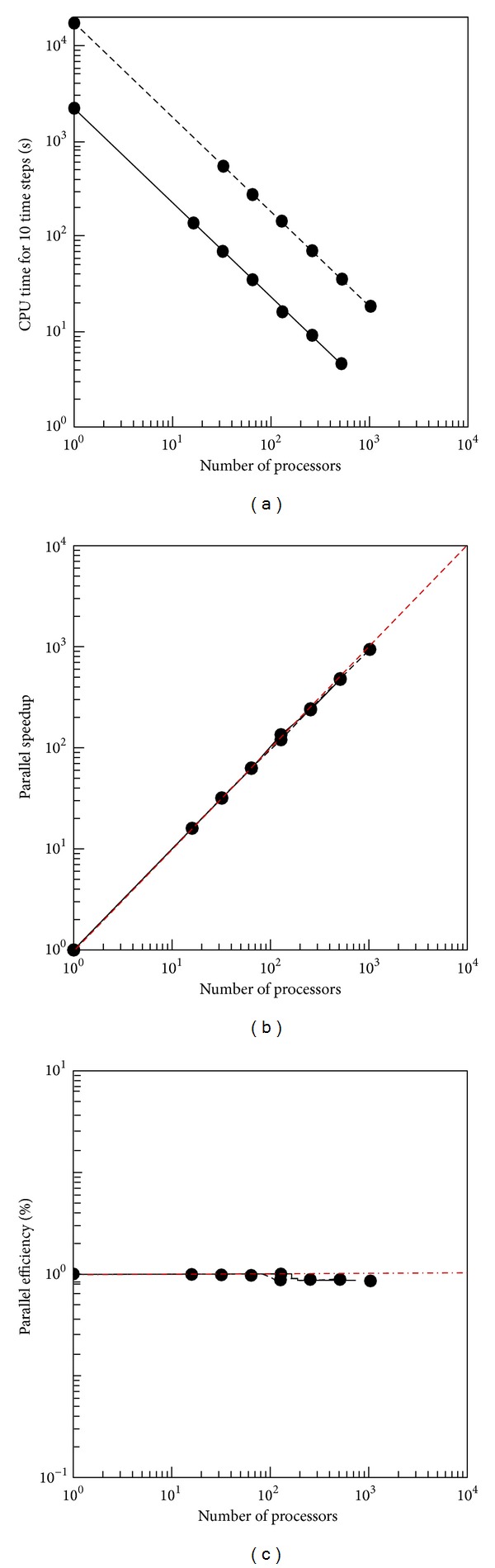
Parallel scaling analysis for the solution of the Euler hydrodynamics system (21) on two different problem sizes: (a) CPU time for 10 time steps, (b) speedup, and (c) efficiency; solid line with symbols: 128^3^; dashed line with symbols: 256^3^. The red dashed line indicates perfect or ideal values.

If a uniform grid is used, then the subdomains in each direction will contain equal number of grid points. However, for a nonuniform grid, the the division locations between the subdomains need to be selected to provide good load balancing or an equivalent amount of work for each processor in each time step. Hence, for the purpose of a scaling analysis, Figure 7 illustrates the CPU time, parallel speedup, *S*_*p*_ = *T*_serial_/*T*_parallel_, and the parallel efficiency, *E*_*p*_ = *S*_*p*_/*p*, where *T*_serial_, *T*_parallel_, and *p* are the CPU time for serial and parallel runs and the number of processors, respectively. The scaling analysis is presented for the numerical solutions of the Euler hydrodynamic system presented before; (21) diagnostics for two different problem sizes are presented: one is with 128^3^, while the other is with 256^3^ number of points. The simulations were conducted on the Glenn cluster at Ohio Supercomputing. Due to the high memory requirements of the code, the lowest number of processors on which a 128^3^ simulation can be run is 16, while the corresponding number for the 256^3^ simulation is 32. In order to present a complete scaling analysis, that is, to calculate speedup and efficiency, it is assumed that these quantities are ideal up to 16 and 32 processors for the 128 and 256^3^ simulations, respectively.

Figure 7 shows the simulation time for 10 time steps on a log scale, where the point corresponding to a single processor was in fact extrapolated from the nearest point assuming ideal efficiency (100%). The CPU time decreases linearly with the number of processors which is encouraging. On the same figure speedup and efficiency are close to ideal (red dashed line), with efficiency values ranging between 94% and 100%.

### 4.3. Euler System of Equations: Richtmyer-Meshkov Instability

Euler equations of gas dynamics are given by

(21) $$\begin{array}{c} {\left(\rho \right)}_{t}+\nabla \cdot \left(\rho \upsilon \right)=0,\\ {\left(\rho \upsilon \right)}_{t}+\nabla \cdot \left[\left({\rho \upsilon \upsilon }^{T}\right)+pI\right]=0,\\ {\left(E\right)}_{t}+\nabla \cdot \left[\left(\frac{\gamma }{\gamma -1}p+\frac{1}{2}\rho v^{2}\right)\upsilon \right]=0. \end{array}$$

Here *ρ* and *E* are scalar quantities representing the mass density and total internal energy, respectively. **υ** = (*u*,*v*,*w*)^*T*^ is the velocity field with* Euclidean* norm *υ* ^2^ : = ||**υ**||^2^. The pressure, *p*, is coupled to the total internal energy, *E* = (1/2)*ρυ* ^2^ + *p*/(*γ* − 1).

The evolution of the Richtmyer-Meshkov instability (RMI) [25, 26] is considered in this section. RMI arises when a shock passes through an interface between two fluids of widely ranging densities. A generic feature of these systems, as is the case for fluid turbulence in general, is the existence of fluctuations on multiple length scales. Three-dimensional simulations of the reshocked RMI modeled after the Mach 1.21 experiment of Collins and Jacobs [27] are presented in the present work. The simulations use the 3rd-order CWENO reconstruction method without diagonal smoothing (to avoid excess dissipation resulting from it) using 1024 × 512 × 512 grid points on a domain of 17.8 × 8.9 × 8.9 cm^3^. For test purposes and in order to have a higher resolution, the domain size here in these simulations is more than 50% smaller in the *x*-direction as compared to experiments.

**Figure 8:**
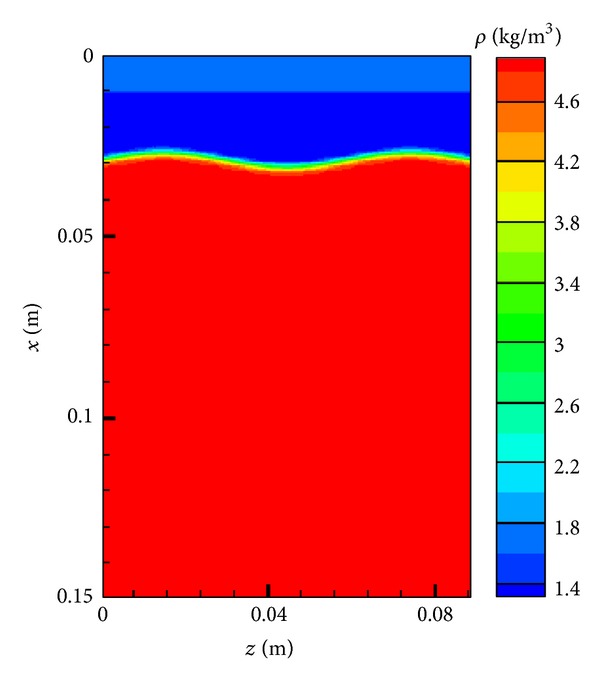
Instantaneous contours of initial density across centerline *y*-direction (*Y* = 0) at *t* = 0 ms.

The initial conditions were adapted from the Mach 1.21 air/SF_6_ experimental shock tube configuration of Collins and Jacobs [27]. The adiabatic exponent *γ* = 1.24815 corresponding to an air mixture was used. The ratio of densities is given by *ρ*_SF_6__/*ρ*_air_ = 4.063. The initial sinusoidal interface *η*(*y*, *z*) = *a*_*o*_  sin(2*πy*/*λ*)  sin(2*πz*/*λ*) had preshock amplitude *a*_*o*_ = 0.2 cm and wavelength *λ* = 5.933 cm. An initial diffusion layer thickness of *δ* = 0.5 cm was used [28], where the thickness function is *S*(*x*, *y*, *z*) = 1 if *d* ≤ 0, = exp⁡(−*α*|*d*|^8^) if 0 < *d* < 1 and 0 otherwise. *d* = (*x*_*s*_ + *η*(*y*, *z*) + *δ* − *x*)/(2*δ*), and *α* = −ln⁡*β* (*β* is machine zero).  Figure 8 shows the initial condition in terms of density for this case. See [28] for a 2D implementation of these initial conditions. 

The following boundary conditions were used: (a) inflow at the test section entrance in the streamwise *x*-direction; (b) reflecting at the end wall of the test section in the streamwise direction; and (c) being periodic in the *y*- and *z*-directions corresponding to the cross-section of the test section. The reflecting boundary condition is implemented by reversing the normal component of the velocity vector: *u*(*x*, *t*) = −*u*(*x*, *t*) at *x* = 17.8 cm (maximum in the streamwise direction) and at the ghost points, which is exact and does not generate spurious noise [28].

**Figure 9:**
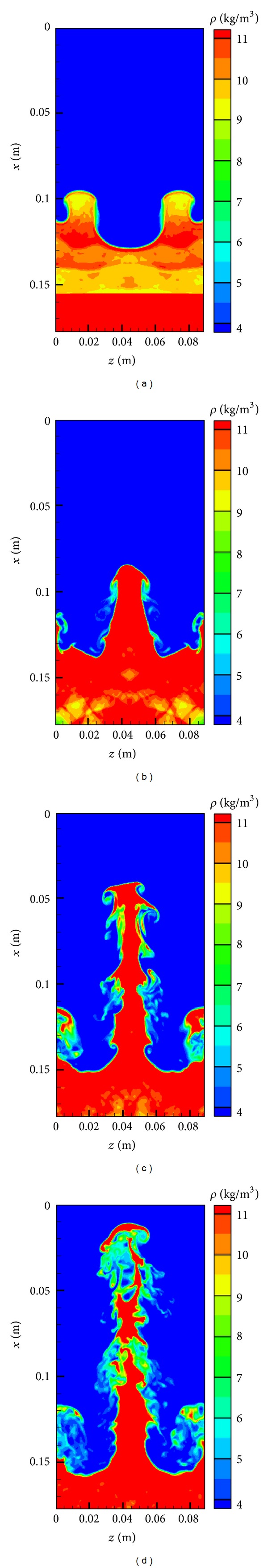
Instantaneous contours of density across centerline *y*-direction (*Y* = 0) at (a) *t* = 1 ms, (b) *t* = 2 ms, (c) *t* = 3 ms, and (d) *t* = 4 ms.

**Figure 10:**
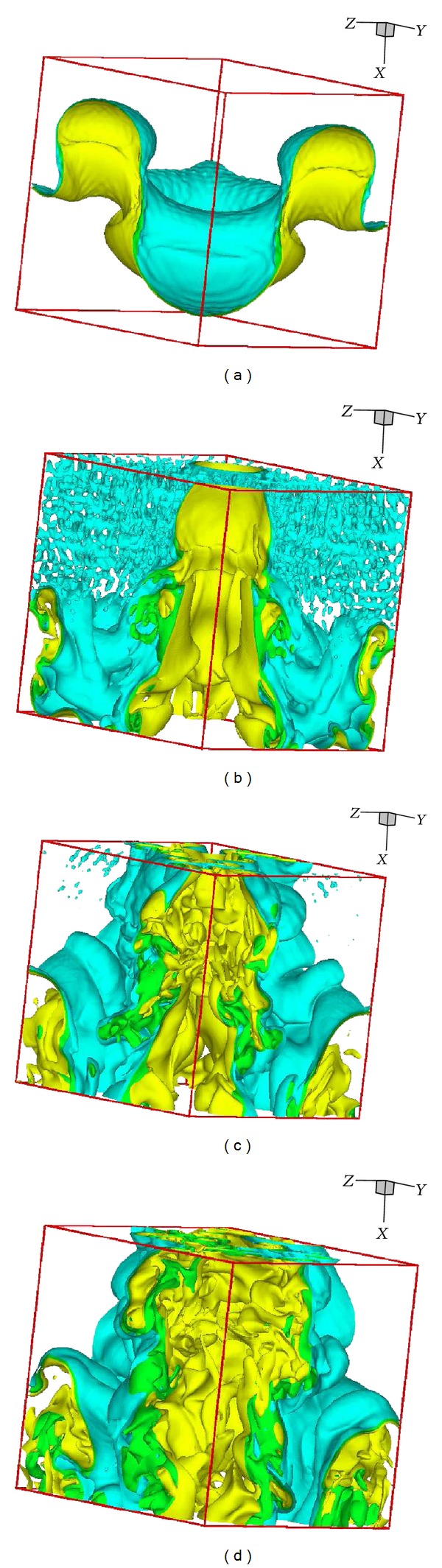
Instantaneous isosurfaces of density (*C* = 7 and *C* = 9) on a subdomain of size 4.5 × 4.5 × 4.5 cm^3^, (a) *t* = 1 ms, (b) *t* = 2 ms, (c) *t* = 3 ms, and (d) *t* = 4 ms.

Figures 9 and 10 show the instantaneous contour slices of density and isosurfaces of density, respectively, at times given by *t* = 1 ms, *t* = 2 ms, *t* = 3 ms, and *t* = 4 ms. As the RMI instability develops, spikes of SF_6_ fall into the air. Following this initial growth, the spikes roll-up and additional complex structures begin to appear. The results presented here are qualitatively similar to those of other studies, for example, [28].

### 4.4. Ideal Magnetohydrodynamic (MHD) Equations: Orszag-Tang Vortex System

The system of equations for ideal magnetohydrodynamics (MHD) is given by

(22) $$\begin{array}{c} {\left(\rho \right)}_{\text{t}}+\nabla \cdot \left(\rho \upsilon \right)=0, \end{array}$$

(23) $$\begin{array}{c} {\left(\rho \upsilon \right)}_{\text{t}}+\nabla \cdot \left[\left({\rho \upsilon \upsilon }^{\text{T}}\right)+\left(p+\frac{1}{2}B^{2}\right)I-{BB}^{T}\right]=0, \end{array}$$

(24) $$\begin{array}{c} {\left(B\right)}_{\text{t}}-\nabla \times \left(\upsilon \times B\right)=0, \end{array}$$

(25) $$\begin{array}{c} {\left(E\right)}_{\text{t}}+\nabla \cdot \left[\left(\frac{\gamma }{\gamma -1}p+\frac{1}{2}\rho \upsilon^{2}\right)\upsilon -\left(\upsilon \times B\right)\times B\right]=0. \end{array}$$

Here *ρ* and *E* are scalar quantities representing the mass density and total internal energy, respectively, **υ** = (*u*, *υ*, *ω*)^*T*^ is the velocity field with *Euclidean* norm *υ* ^2^≔||**υ**||^2^, and **B** = (*B*_1_, *B*_2_, *B*_3_)^*T*^ and *B* ^2^≔||**B**||^2^ represent the magnetic field and its norm, respectively. The pressure, *p*, is coupled to the total internal energy, *E* = (1/2)*ρυ* ^2^ + *p*/(*γ* − 1) + (1/2)*B* ^2^. Furthermore, the system of MHD equations is augmented by the solenoidal constraint; that is, if the condition ∇·**B** = 0 is satisfied initially at *t* = 0, then by (24) it remains invariant in time.

**Figure 11:**
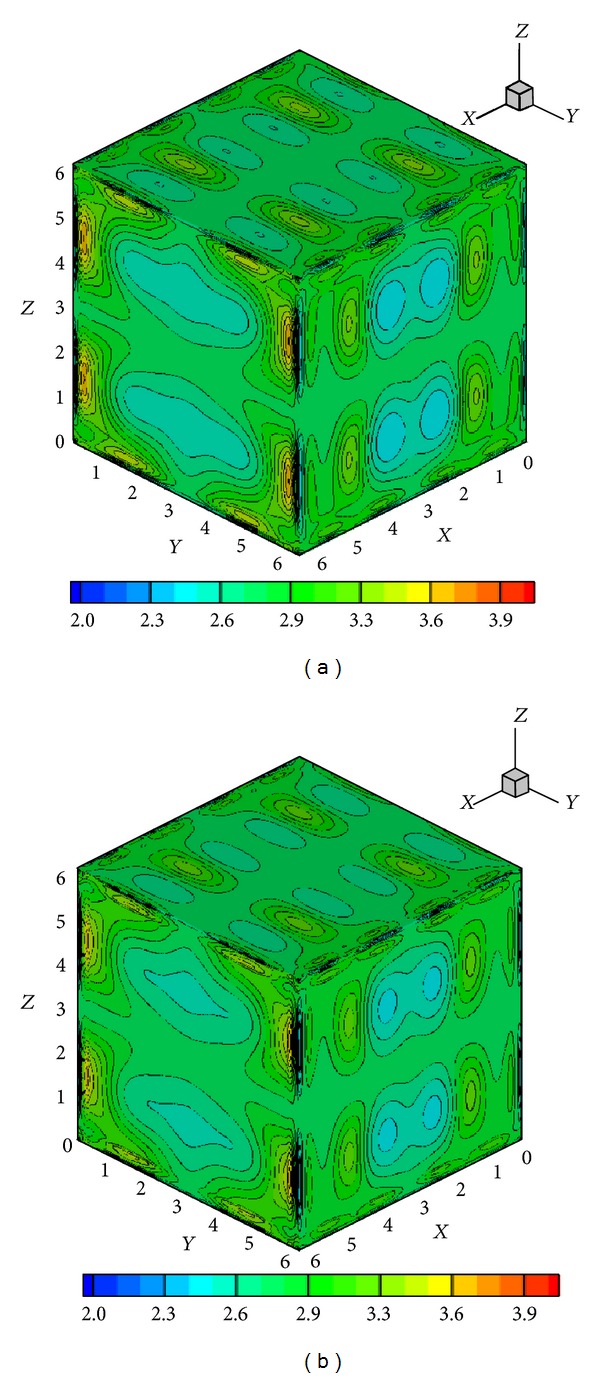
Instantaneous contours of density across *x*-, *y*-, and *z*-direction centerlines ( = *π*) at *t* = 0.2 for two different grid sizes 128^3^ (a) and 256^3^ (b).

The 3D problem, that is investigated here, is a 3D MHD problem, the Orszag-Tang-type problem [29]. The evolution of the vortex system involves the interaction between several shock waves traveling at various speed regimes [30, 31], which makes the problem especially attractive for numerical experiments. The initial data for this problem are the following:

(26) ρ=γ2,p=γ,u=(−sin⁡ysinz, sinx  sinz, 0),B=(−sin⁡ysinz, sin2x  sinz, sin2x  sin⁡y)

with 0 ≤ *x*, *y*, *z* ≤ 2*π*, where *γ* = 5/3. Again, grid independence is demonstrated in Figure 11 through density contours on slices across the centerlines planes on coarse (128^3^) and fine grids (256^3^). The results presented here in this section are those computed on the 256^3^ grid using the CWENO reconstruction without diagonal smoothing.

**Figure 12:**
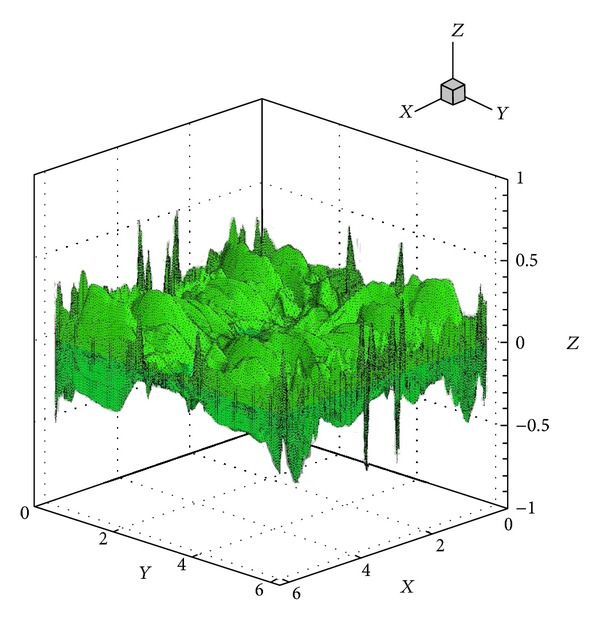
Instantaneous surface plot of the divergence of the magnetic field (∇·**B**) on all the *Z*-surfaces at *t* = 0.5 for the solution of the Orszag-Tang system.

**Figure 13:**
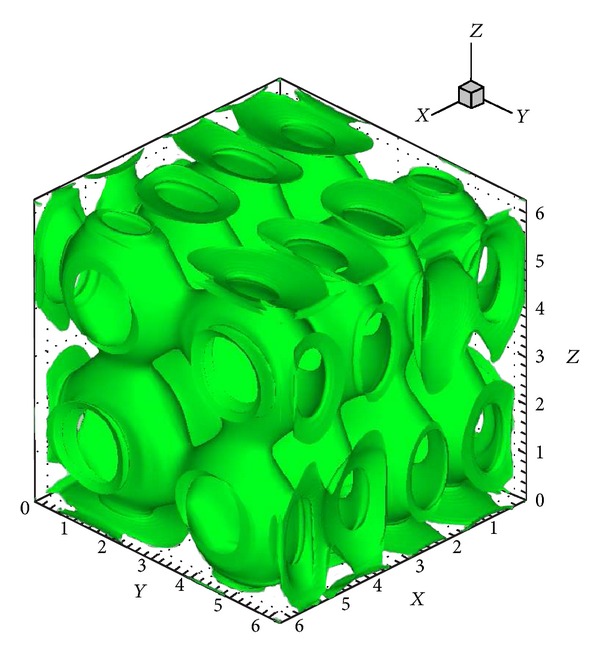
Isosurface of the density at a value *ρ*_*C*_ = 3.0 at *t* = 0.2.

**Figure 14:**
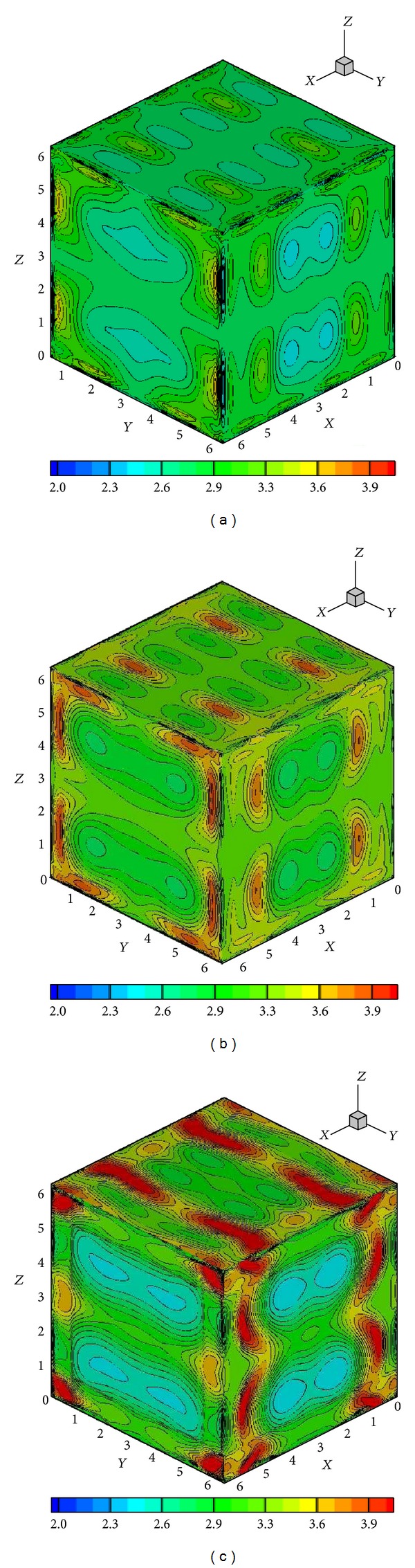
Instantaneous contours of density across *x*-, *y*-, and *z*- direction centerlines ( = *π*) at (a) *t* = 0.2, (b) *t* = 0.4, and (c) *t* = 0.8 (grid: 256^3^).

**Figure 15:**
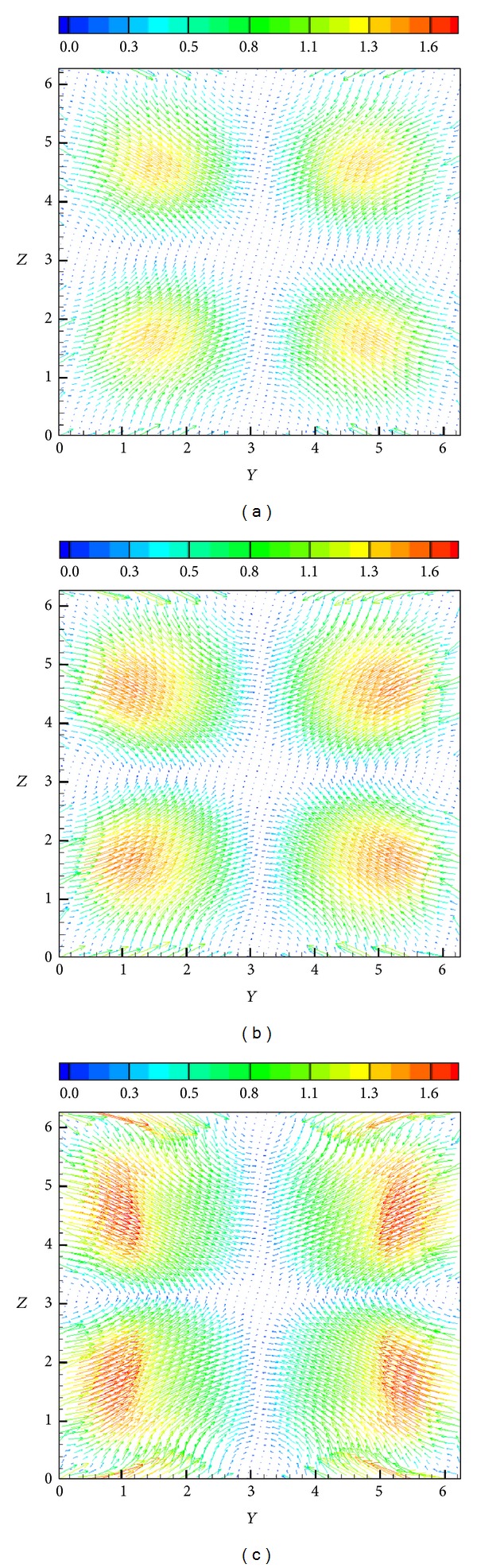
Instantaneous slices across the *x*-direction centerline ( = *π*) showing vectors of the 2D magnetic field (*B*_*y*_, *B*_*z*_) colored by the magnitude of the magnetic field at (a) *t* = 0.2, (b) *t* = 0.4, and (c) *t* = 0.8 (grid: 256^3^).

A way of demonstrating the accuracy of a numerical method is to determine whether the solenoidal constraint ∇·**B** = 0 is maintained throughout the simulation. Since ∇·**B** = 0 initially, theoretically it should remain so throughout the simulation. However, the accumulation of numerical errors can usually lead to nonphysical phenomenon known as magnetic monopoles (when ∇·**B** is not equal to 0). The schemes presented here when first introduced in [21] in 1D and 2D frameworks did not require an explicit enforcement of the solenoidal constraint for producing stable and reasonably accurate solutions, and hence no such treatment is used here either.  Figure 12 shows the surface plots of ∇·**B** on all the *Z*-surfaces at a certain instant in time. The maximum value of the magnitude of ∇·**B** at this instant is 0.41, which is actually representative of the entire simulation. Although these simulations did not show any kind of instabilities, the divergence values are reasonably large for such computations and go to show that in 3D some divergence treatment [32, 33] might be necessary.  Figure 13 shows a density isosurface, and Figure 14 shows contours of density on three slices across the *x* = *y* = *z* = *π* planes.  Figure 15 shows the 2D magnetic field vector colored by magnetic field magnitude. These results are similar to those of previous studies [32, 33] and demonstrate the ability of such higher-order central schemes to resolve the shocks that the vortex system develops while maintaining the simplicity and ease of implementation typical of this black-box type of finite difference schemes.

## 5. Conclusions

Extensions of the semi-discrete schemes of Balbás and Tadmor [21] to 3D are presented and tested for the first time in this paper. The numerical test cases chosen include evolution of (a) a single scalar equation or an inviscid Burgers equation, (b) the Richtmeyer-Meshkov instability (RMI) using Euler hydrodynamic equations, and (c) the Orszag-Tang vortex system using ideal magnetohydrodynamic equations. Grid independence was demonstrated for two of the three cases presented here. For the single scalar equation test case, our 3rd-order results exhibited more dissipation than those with corresponding 2nd-order schemes with minmod reconstructions. Parallel scaling analysis showed almost ideal efficiencies and speedups based on the assumption that they hold ideal values up until 16 processors. The results obtained with these schemes for the Richtmeyer-Meshkov instability and the Orszag-Tang vortex system confirm the ability of this type of solver to approximate the discontinuous solutions of Eulerian gas dynamics and ideal magnetohydrodynamics equations.
